# Isorhamnetin as a promising agent for reducing diabetes-induced cardiac damage: insights into antioxidant and enzyme regulatory mechanisms

**DOI:** 10.1007/s11033-025-10941-1

**Published:** 2025-10-16

**Authors:** Esam Qnais, Abdelrahim Alqudah, Omar Gammoh, Yousra Bseiso, Mohammed Wedyan, Badriyah S. Alotaibi, Alaa A. A. Aljabali, Taher Hatahet

**Affiliations:** 1 Department of Biology and Biotechnology, Faculty of Science, The Hashemite University, Zarqa, Jordan; 2 Department of Clinical Pharmacy and Pharmacy Practice, Faculty of Pharmaceutical Sciences, The Hashemite University, Zarqa, Jordan; 3 Department of Clinical Pharmacy and Pharmacy Practice, Faculty of Pharmacy, Yarmouk University, Irbid, Jordan; 4 Department of Pharmaceutical Sciences, College of Pharmacy, Princess Nourah bint Abdulrahman University, P.O. Box 84428, Riyadh, 11671 Saudi Arabia; 5 Department of Pharmaceutics and Pharmaceutical Technology, Faculty of Pharmacy, Yarmouk University, Irbid, 21163 Jordan; 6 North Wales Medical School, Bangor University, Brigantia Building, Penrallt Road, Bangor, Gwynedd, LL57 2AS Wales, UK

**Keywords:** Isorhamnetin, Diabetes mellitus, Cardioprotection, Oxidative stress, STZ-induced diabetic rats

## Abstract

**Purpose:**

This study assessed the protective effect of Isorhamnetin in diabetic rats induced by streptozotocin (STZ).

**Methods:**

Male Wistar rats were randomly divided into five groups. Normal control, Diabetic control, Low dose of Isorhamnetin – 50 mg/kg, High dose of Isorhamnetin – 150 mg/kg, and Metformin – 200 mg/kg. Diabetes was induced by a single intraperitoneal injection of STZ and treatment was given orally for a period of 21 days. The parameters that were observed in this study were oxidative markers, ATPase and phosphatase activities, p53 and VCAM-1gene expression, myocardial injury markers and histopathology.

**Result:**

Diabetic rats increased the level of MDA, decreased antioxidant enzyme catalase, SOD, GPx, and GST activity, decreased ATPase activities of Na+/ K+-ATPase, Ca2+/Mg2+- ATPase, and Mg2+-ATPase, increased the expression of p53 and VCAM-1 gene compared to normal control. Low and High dose of Isorhamnetin significantly decreased the MDA level, increased the antioxidant enzyme activity, ATPase, and Na+/K+-ATPase activity compared to diabetic rats and this activity was similar to metformin. Isorhamntenin decreased the p53 and VCAM gene concisely to the diabetic group, increased AST and ALTand reduced the CK-MB and cardiac troponins. Histopathological study showed that reduced the muscle fiber degeneration and congestion pattern of heart congestion.

**Conclusion:**

Isorhamnetin plays a cardioprotective effect in diabetic rats by reducing oxidative stress, increased antioxidant defense, restored and enzyme activity of ATPase and reduced inflammation and apoptosis. Hence, Isorhamnetin can be used as a promising multi-target drug for diabetes-induced cardiac and injury.

## Introduction

Diabetes mellitus is a chronic metabolic disorder characterized by sustained hyperglycemia, resulting from impaired insulin production, insulin action, or both [1]. The global prevalence of diabetes has grown dramatically, presenting significant public health challenges [2]. Chronic hyperglycemia is linked to a host of complications that affect multiple organs, including the cardiovascular system [3]. These complications are often exacerbated by underlying oxidative stress, inflammation, and metabolic disturbances, which highlights the importance of developing therapeutic agents that target these mechanisms [4].

The traditional concept that oxidative stress is one of the primary theoretical biological mechanisms leading to diabetes and its complications. This is created due to an imbalance of the production of reactive oxygen species (ROS) versus the ability of the body to neutralize these ROS by antioxidant defenses, which results in damage mainly at a cellular level [5]. Loss of activity in the antioxidant enzyme superoxide dismutase was accompanied by enhanced lipid peroxidation and depleted levels of reduced glutathione, typical hallmarks of oxidative stress during diabetes [6]. Aberrant regulation of enzymes such as catalase (CAT), superoxide dismutase (SOD), glutathione peroxidase (GPx), and Glutathione-S-transferase (GST) have been reported in diabetic hearts, which contributes to this tissue damage and dysfunction [7].

The cardiac ATPase enzymes Na^+^/K^+^-ATPase, Ca^2+^/Mg^2+^-ATPase, and Mg^2+^-ATPase are critical in the maintenance of ion gradients, energy metabolism, and overall myocardial function [8]. Defects in these enzymes induced by diabetes causes a disorder in ion regulation such as calcium uptake and release from mitochondria lead to abnormal myocardial contractility that favors the development of diabetic cardiomyopathy [9]. Therefore, alleviating the cardiac dysfunction associated with diabetes by restoration ATPase activities might be an interesting therapeutic approach.

Moreover, cellular stress and inflammation caused by diabetes play a crucial role in the p53 and vascular cell adhesion molecule-1 (VCAM-1) expression. Diabetic conditions lead to upregulation of p53 (a tumor suppressor gene) in the myocardium inducing apoptosis [10]. VCAM-1, a crucial endothelial cell adhesion molecule for which binding increases leukocyte adhesion, plays an important role in diabetic cardiovascular complications [11]. Together these factors further underscore the need to modulate not only apoptotic but also inflammatory pathways for optimal cardiovascular outcome in diabetes.

Activities of phosphatases, such as alkaline phosphatase (ALP) and acid phosphatase (ACP), are also markedly affected under the diabetic states, leading to the impairment of cell metabolism and tissue integrity [12]. Moreover, high concentrations of the cardiac injury markers: creatine kinase-MB (CK-MB); alanine transaminase (ALT); aspartate transaminase (AST) and cardiac troponins, are indicative of myocardial injury under the conditions such as diabetes mellitus than those seen in heart diseases [13]. These changes in phosphatase activity and cardiac injury markers provide valuable information of the degree of myocardial damage as well as therapeutic interventions efficacy.

Isorhamnetin is a flavonoid found in medicinal plants and has been shown to have potent antioxidant, anti-inflammatory, and cardioprotective effects [14]. Its ability to scavenge ROS, ameliorate endogenous antioxidant defenses, and suppress pro-inflammatory mediators render it an appealing therapeutic agent for the treatment of Diabetic mellitus and its complications [15]. Studies have indicated that isorhamnetin can effectively reduce lipid peroxidation and enhance the activity of antioxidant enzymes such as SOD, CAT, and GPx, suggesting its therapeutic potential in alleviating oxidative stress-related complications in diabetes [16]. Moreover, isorhamnetin has been shown to modulate inflammatory pathways, reducing pro-inflammatory cytokine levels and suppressing key inflammatory mediators involved in diabetic complications [17]. These properties make isorhamnetin a potential candidate for mitigating cardiovascular damage associated with diabetes and improving myocardial function.

The current study explores the cardioprotective effects of Isorhamnetin in streptozotocin (STZ)-induced diabetic rats and its influence on oxidative stress markers, ATPase and phosphatase enzyme activities, gene expression levels of p53 and VCAM-1 as well as cardiac appearance for myocardial injury. The performance of Isorhamnetin was compared with the reference Metformin (a well-known antidiabetic drug).

## Materials and methods

### Induction of diabetes in rats

Male Wistar albino rats, aged 8–9 weeks and weighing 180–200 g, were used to induce diabetes using an adapted protocol from Alshehri [18]. The rats were obtained from an approved breeder and were allowed at least one week to acclimate to the laboratory environment before beginning the experimental protocol in order to mitigate any changes in stress due to environmental disruptions. All animals were maintained in groups of four rats per cage under controlled conditions (temperature 22 ± 2 °C, humidity 55 ± 5%, light/dark cycle = 12/12 h). The goal was to reduce environmental variation using this setup. Rats were provided ad libitum access to standard pellet chow and tap water.

Here, rats were fed 20% fructose in drinking water for one week along with pellet diet to create a pre-diabetic state and to induce insulin resistance. Thus, it provided a model of early metabolic changes contributing to the development of Type 2 diabetes in order that the subsequent effects of streptozotocin (STZ) could be evaluated with respect to their significance in provoking a state identical to diabetes development under insulin resistance. In contrast, rats in the normal control group received regular tap water and a similar pellet diet but without fructose supplementation.

One week after this treatment period, all rats had a 12 h access to water but no food (water ad libitum) for fasting conditions important for diabetes induction. Fasting promotes STZ´s action on pancreatic beta cells, diminishing the interference of glucose levels during the STZ application. Diabetic rats were induced with diabetes by an intraperitonial injection of STZ 40 mg/kg body weight. STZ was purchased from Sigma-Aldrich (UK) and freshly dissolved into cold pH 7.4 sodium citrate buffer to preserve the stability and bioactivity of STZ at time of administration. STZ has been known as a diabetogenic agent for its selective destructive action on pancreatic beta cells and is commonly used to induce sustained hyperglycemia in an animal model that mimics human diabetes pathology.

The dose of STZ (40 mg/kg) used in this study was determined by previous reports, which identified this as the minimum dose with reproducible induction of hyperglycemia, and also myocardial injury compatible with diabetic cardiomyopathy. This dose of STZ has been shown to result in sustained hyperglycemia associated with increased cardiac oxidative stress, apoptosis and histopathologic abnormalities including degeneration and vacuolization of myocardial fibers, consistent with previous data from our laboratory validating the use of this model for the study of diabetes-induced cardiac damage [19, 20].

Fasting blood glucose levels were measured for diabetic confirmation three days post-STZ injection. Blood glucose was determined by the glucometer (Accu-Chek, Roche) with a sample of tail vein blood. Rats were considered to have diabetes if the fasting blood glucose was elevated at ≥ 250 mg/dL, a value typical of severely uncontrolled diabetes and thus most ideal for use in experimental groups. Only animals that failed to achieve this cutoff were excluded in order to maintain the uniformity of diabetic status among all groups throughout the study.

## Experimental design and animal treatment

### Study setup

The authors followed guidelines of the ARRIVE (Animal Research: Reporting In Vivo Experiments) to increase the reporting transparency and reproducibility in animal experiments. The experimental procedure was permitted by Institutional Animals Care and use Committee (IACUC) at Hashemite University, Zarqa/Jordan/protocol no. 51/3/2024/2025. This approval meant that all procedures used in this study followed the strict guidelines required by the ethical standards of animal use for research, with the goal of minimizing animals’ pain and distress throughout the study.

## Animal grouping and treatment

Then forty male Wistar rats were stratified by body weight and then divided to 5 different groups including;

- Group I (Normal Control): non-diabetic rats not induced with diabetes or treatment.

• Group II (Diabetic Control): Diabetic rats of age 8 weeks for normal diet without any treatment.

- Group III (Low Dose Isorhamnetin): Diabetic rats given low dose of Isorhamnetin at 50 mg/kg body weight, Sigma-Aldrich, UK.
- Group IV: Diabetes rats treated with a high amount of isorhamnetin (150 mg/kg bwt).
- Group V (Metformin Treatment): Diabetic rats maintained with metformin at a dose of 200 mg/kg body weight that could serve as a control group for the standard diabetic treatment.

Treatment was via oral intubation daily over the course of the study to ensure consistent and exact dosing. This treatment lasted 21 days, with close monitoring and recording of each administration to accurately evaluate adverse effects.

Doses of isorhamnetin (50, 150 mg/kg) were selected according to earlier published studies that showed strong anti-inflammatory, antioxidant and cardiac protective effects of isorhamnetin in rodent models with low toxicity profile [21]. The dose (50 mg/kg) is the lower dose required for therapeutic efficacy, whereas the latter (150 mg/kg) was used to analyze a potential robust and dose-dependent effect. The duration of treatment for 21 days was selected because this is long enough to observe the evolution of STZ-induced diabetic cardiomyopathy and the effect of isorhamnetin in terms oxidative stress, gene expression, and myocardial injury markers [19].

## Ethical aspects and monitoring

All animals were acclimatized to standard laboratory conditions of 22 ± 2 °C, 55 ± 5% humidity and a light–dark cycle (12 h/12 h) during the experimental study. After model establishment, the rats had eaten and drank freely (except for fasting control), then taken the specified treatment, and were excluded from free access to food before fast. Health check behavior was evaluated by regular health checks such as general well-being (i.e. behaviour, food intake and weight). A rat exhibiting significant weight loss, an abnormal posture or extreme lethargy, was humanely killed in accordance with IACUC protocols.

### Tissue collection and processing

#### Sample collection

At the end of the experimental period (day 22), all rats were euthanized under deep anesthesia using chloroform inhalation to ensure minimal suffering. Following euthanasia, blood samples were collected via cardiac puncture, which provided a reliable method for obtaining a sufficient volume of blood quickly, thus minimizing post-mortem changes. Blood was collected into plain glass tubes for further processing.

## Blood processing

The collected blood samples were centrifuged at 1500 rpm for 10 min to separate the serum from the cellular components. The serum was carefully extracted, aliquoted, and stored at 4 °C until biochemical assays were performed. This low temperature helped to maintain the stability of the serum’s biochemical constituents until analysis.

### Heart tissue Preparation

After blood collection, each rat’s heart was excised, rinsed thoroughly with ice-cold normal saline to remove residual blood, and dried on filter paper. The hearts were weighed to assess morphological changes potentially related to treatment or disease status. Subsequently, the heart tissues were homogenized in 0.1 M potassium phosphate buffer (pH 6.5) using a motor-driven homogenizer to ensure a consistent homogenate suitable for biochemical evaluations. The homogenization provided uniform samples for enzyme assays and oxidative stress biomarker analysis.

### Homogenate processing

After 15 min centrifugation of homogenates at 4000 rpm and at 4 °C, a clear supernatant was obtained from each which was then used for various biochemical determinations like enzyme activity, oxidative stress markers etc. The supernatant was kept on ice while processing to minimize any potential break down of heat sensitive components.

### Biochemical assays

#### Cardiac oxidative stress biomarker assays

##### Measurement of malondialdehyde (MDA) levels

Levels of malondialdehyde (MDA), a marker of lipid peroxidation were determined by the thiobarbituric acid reactive substances (TBARS) method. Regarding this assay, 2 mL heart tissue sample was homogenate in 2 mL of 0.7% TBA and then precipitated with 1 mL for Trichloroacetic acid (TCA). The solution was incubated in a water bath at 100 °C during 20 min, then allowed to cool down to room temperature and finally centrifuged at 4000 rpm for 10 min. The absorbance of developing supernatant was measured at 540 nm using determined distilled water as a reading blank. MDA content (nmol/mg protein).

### Superoxide dismutase (SOD) activity was measured

The activity of superoxide dismutase (SOD) was measured using a standard method. In brief, 0.5 mL of heart homogenate was diluted with 5 mL distilled water and then mixed with carbonate buffer (pH 10.2; final concentration: 0.05 M) in a total volume of 2.7 ml for assay. 0.3 mL of a 0.3 mM epinephrine was adding to the reaction, and after initialization (t = 0) 480 nm OD readings were taken every 30 s for 150 s. The activity of SOD was measured as inhibition of epinephrine autoxidation and expressed in unit’s/mg protein.

### Assays for Glutathione-S-Transferase (GST) activity

Glutathione-S-transferase (GST) activity was measured using a standard method. The reaction mixture consisted of 0.1 M potassium phosphate (pH 7.5), 1 mM GSH, and 1 mM CDNB (or DCNB) plus a specified solvent at a maximum of 1% ethanol concentration. The reaction was started by the addition of substrate, and absorbance at 340 nm was measured for 2 min. The enzymatic activity was defined as µmol of conjugated product per minute per mg protein.

### Measurement of catalase and glutathione peroxidase activities

Catalase activity was determined by measuring the decrease in hydrogen peroxide absorbance at 240 nm in a reaction mixture containing 0.1 mL of heart tissue homogenate and 2.9 mL of 0.036% H₂O₂. Glutathione peroxidase (GPx) activity was assessed by incubating the heart homogenate with reduced glutathione (GSH) and hydrogen peroxide, followed by reaction with DTNB, and the absorbance was measured at 412 nm using a spectrophotometer.

### Molecular gene expression

For the assessment of gene expression of p53 and VCAM-1, total RNA was extracted from cardiac tissues using TRIzol reagent. Quality control of the RNA was conducted by measuring absorbance ratios (260/280 nm) to confirm the absence of protein contamination. RNA was then converted to complementary DNA (cDNA) using the RevertAid First Strand cDNA Synthesis Kit. Gene expression analysis was performed using the 7500 Fast Real-Time PCR System (Applied Biosystems), utilizing SYBR Green for detection. The target genes were p53 and VCAM-1, with GAPDH serving as the housekeeping gene.

### Histopathological examination

Heart tissue sections were fixed in 10% neutral buffered formalin, embedded in paraffin, and cut into 4 μm sections. Sections were stained with hematoxylin and eosin (H&E) to visualize structural details under a Leica ICC5 light microscope. All evaluations were performed by a histopathologist blinded to the treatment groups.

Histopathological analysis using H&E staining has been used to examine cardiomyopathy in experimental models of T2D. This approach facilitates not only the visualization of key histopathological features associated with diabetes-induced cardiac injury, such as myofiber degeneration and vacuolization, nuclear pyknosis and vascular congestion but also the quantification apoptosis and fibrosis [19].

### Statistical analysis

Data were presented as mean ± standard deviation (S.D.) for each group (*n* = 8). Statistical comparisons among groups were performed using one-way analysis of variance (ANOVA) followed by Tukey’s post-hoc test to determine significant differences between the control and treatment groups. A p-value of < 0.05 was considered statistically significant. Statistical analysis was performed using GraphPad Prism software, Version 5.0.

## Results

### Effect of Isorhamnetin on activities and levels of cardiac redox stress biomarkers in STZ-induced diabetic rats

**Fig. 1:**
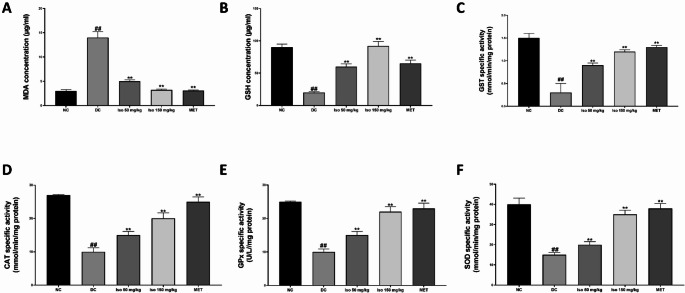
Isorhamnetin modulates heart redox stress biomarkers in streptozotocin-induced diabetic rats. Each value is a mean of eight determinations ± SD. ## *p <* 0.01 vs. NC, ** *p <* 0.01 vs. DC. NC: Normal Control, DC: Diabetic Control, MET: Diabetic rats administered 200 mg/kg of metformin, MDA: Malondialdehyde, GSH: Reduced glutathione GST: Glutathione-S-Transferase, CAT: Catalase, GPx: Glutathione Peroxidase, and SOD: Superoxide dismutase

Figure 1 presents the effect of Isorhamnetin on the levels of oxidative stress markers and antioxidant enzyme activities in the hearts of STZ-induced diabetic rats. In the diabetic untreated group (DC), there was a significant (*p* < 0.01) elevation in lipid peroxidation, indicated by increased MDA levels, compared to the normal control group (NC). This elevated MDA level indicates increased oxidative damage to lipids in cardiac tissue, a hallmark of diabetes-induced oxidative stress.

Concurrently, the activities of key antioxidant enzymes, including Glutathione-S-transferase (GST), catalase (CAT), glutathione peroxidase (GPx), and superoxide dismutase (SOD), were significantly (*p* < 0.01) reduced in the diabetic untreated group (DC), reflecting impaired antioxidant defense mechanisms in the diabetic heart. The level of reduced glutathione (GSH), a critical endogenous antioxidant, was also significantly decreased (*p* < 0.01) compared to the normal control group.

Upon administration of Isorhamnetin at both low (50 mg/kg) and high (150 mg/kg) doses, these changes were significantly (*p* < 0.01) mitigated. The MDA levels decreased, indicating reduced lipid peroxidation, while the activities of GST, CAT, GPx, and SOD were significantly (*p* < 0.01) enhanced in a dose-dependent manner, approaching levels seen in normal control rats. The GSH level also showed a marked increase (*p* < 0.05) compared to untreated diabetic rats. Treatment with metformin (200 mg/kg), used as a standard reference, similarly restored these biomarkers close to normal levels, highlighting the efficacy of Isorhamnetin in mitigating oxidative stress in diabetic hearts.

### Effect of Isorhamnetin on cardiac ATPase activity in the heart of STZ-Induced diabetic rats

**Fig. 2:**
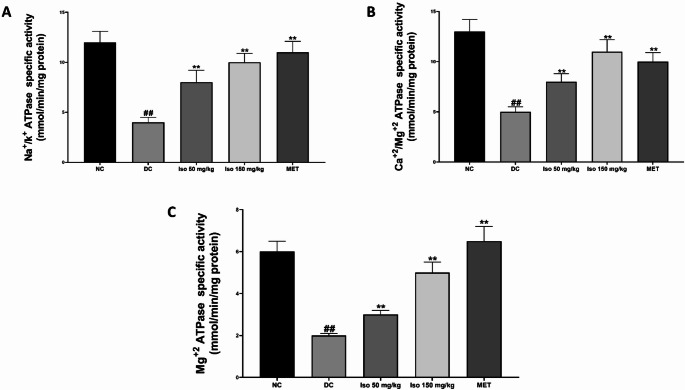
Isorhamnetin improved heart ATPase activities of in streptozotocin-induced diabetic rats. Each value is a mean of eight determinations ± SD. ## *p <* 0.01 vs. NC, ** *p <* 0.01 vs. DC. NC: Normal Control, DC: Diabetic Control, MET: Diabetic rats administered 200 mg/kg of metformin

As depicted in Fig. 2, streptozotocin administration caused a significant (*p* < 0.01) reduction in the activities of cardiac ATPase enzymes, specifically to maintain chemical formulas Na^+^/K^+^-ATPase, Ca^2+^/Mg^2+^-ATPase, and Mg^2+^-ATPase, in the diabetic control group (DC) compared to the normal control (NC). These enzymes are crucial for maintaining ion balance, cellular homeostasis, and energy metabolism in cardiac cells, and their decreased activity is indicative of cardiac dysfunction.

Treatment with Isorhamnetin at both the low and high doses resulted in a significant (*p* < 0.01) restoration of ATPase activity, improving the enzyme levels towards those seen in normal control rats. The effect was dose-dependent, with the high dose showing greater efficacy. Metformin, used as a standard treatment, also significantly (*p* < 0.01) improved ATPase enzyme activity, corroborating the therapeutic potential of Isorhamnetin in restoring ATPase function and maintaining cardiac ion homeostasis in diabetic rats.

### Effect of Isorhamnetin on relative gene expression of p53 and VCAM in the heart of STZ-induced diabetic rats

**Fig. 3:**
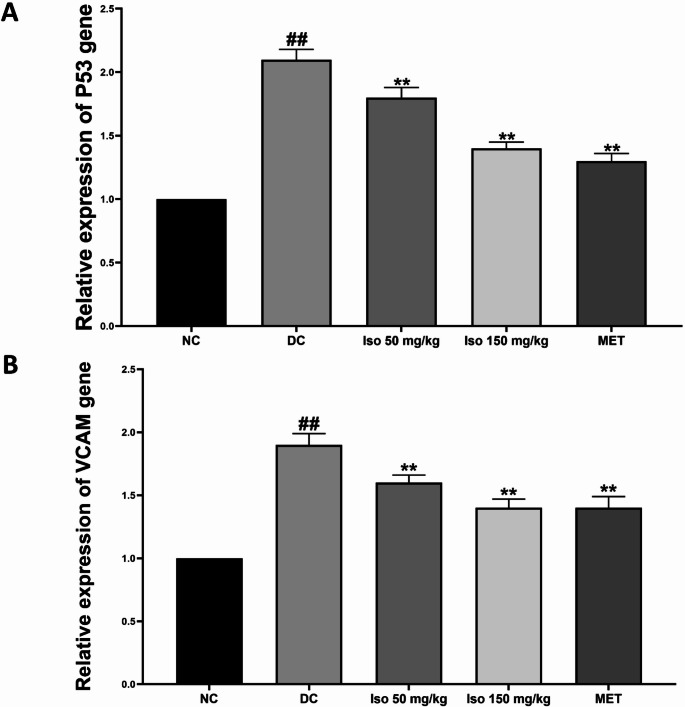
Isorhamnetin upregulates relative gene expression of p53 and VCAM levels of in streptozotocin-induced diabetic rats Each value is a mean of eight determinations ± SD. ## *p <* 0.01 vs. NC, ** *p <* 0.01 vs. DC. NC: Normal Control, DC: Diabetic Control, MET: Diabetic rats administered 200 mg/kg of metformin

Figure 3 illustrates the relative gene expression levels of p53, a tumor suppressor gene, and vascular cell adhesion molecule-1 (VCAM-1), in the hearts of STZ-induced diabetic rats. Both p53 and VCAM-1 were significantly up-regulated (*p* < 0.01) in the diabetic control group (DC) compared to the normal control (NC), as determined using GAPDH as a housekeeping gene for normalization. Elevated p53 expression is indicative of increased cellular stress, apoptosis, or tissue damage, while increased VCAM-1 levels reflect heightened inflammatory and adhesion processes that contribute to vascular complications in diabetic conditions.

Administration of Isorhamnetin at both low and high doses resulted in a significant (*p* < 0.01) down-regulation of p53 and VCAM-1 gene expression, reducing their levels towards those seen in the normal control group. This effect was dose-dependent, with the higher dose providing more pronounced modulation of gene expression. Metformin treatment also resulted in significant (*p* < 0.01) down-regulation of these genes, suggesting that Isorhamnetin may exert similar anti-apoptotic and anti-inflammatory effects in the diabetic myocardium.

### Effect of Isorhamnetin on phosphatase activities in the heart of STZ-Induced diabetic rats

**Fig. 4:**
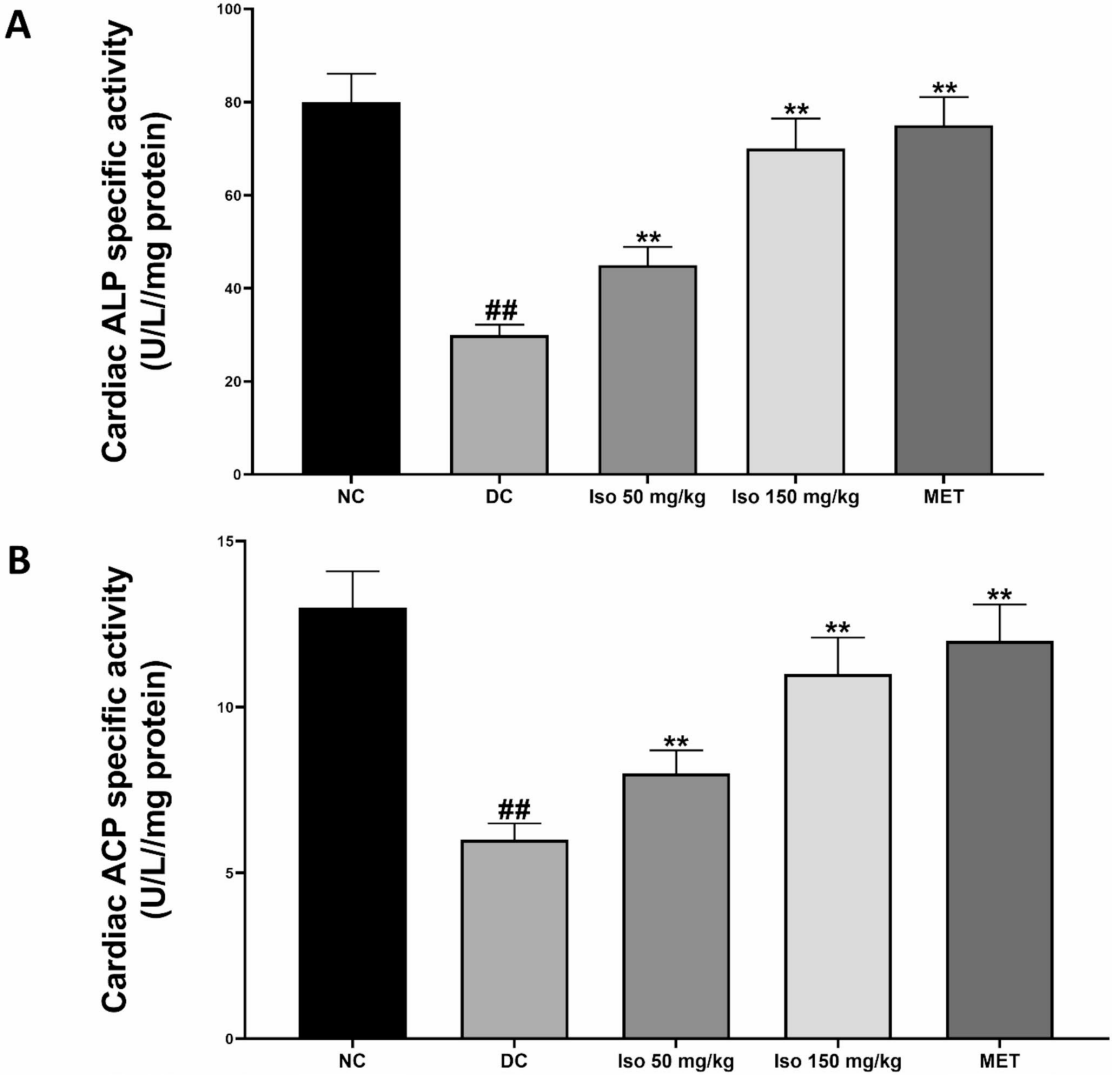
**Isorhamnetin** improved heart phosphatase activities in streptozotocin-induced diabetic rats Each value is a mean of eight determinations ± SD. ## *p <* 0.01 vs. NC, ** *p <* 0.01 vs. DC. NC: Normal Control, DC: Diabetic Control, MET: Diabetic rats administered 200 mg/kg of metformin

The effect of Isorhamnetin on phosphatase activities, specifically alkaline phosphatase (ALP) and acid phosphatase (ACP), in the hearts of STZ-induced diabetic rats is shown in Fig. 4. The diabetic untreated group (DC) exhibited a significant (*p* < 0.01) reduction in the activities of both ALP and ACP compared to the normal control group (NC), indicating impaired phosphatase function in diabetic cardiac tissue.

Treatment with Isorhamnetin significantly (*p* < 0.01) reversed these reductions in phosphatase activities, increasing ALP and ACP activities to near-normal levels, with greater efficacy observed at the higher dose. Similar improvements were observed in the metformin-treated group, highlighting Isorhamnetin’s potential in restoring phosphatase enzyme function in diabetic hearts.

### Effect of Isorhamnetin on cardiac transaminase and serum creatine Kinase-MB activity in STZ-Induced diabetic rats

**Fig. 5:**
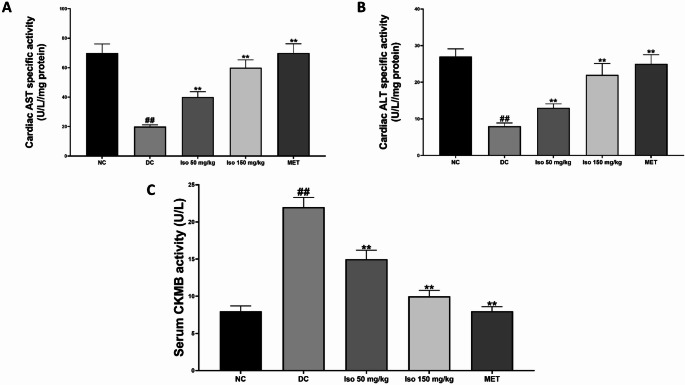
Isorhamnetin modulates heart transaminase and serum creatine kinase-MB (CKMB) activities in streptozotocin-induced diabetic rats. Each value is a mean of eight determinations ± SD. ## *p <* 0.01 vs. NC, ** *p <* 0.01 vs. DC. NC: Normal Control, DC: Diabetic Control, MET: Diabetic rats administered 200 mg/kg of metformin

Figure 5 shows that administration of STZ led to significant (*p* < 0.01) reductions in the activities of cardiac transaminases; alanine transaminase (ALT) and aspartate transaminase (AST), in the diabetic untreated group (DC) compared to the normal control (NC). These enzymes are critical indicators of cellular integrity and metabolic function, and their decreased activity reflects myocardial damage. In contrast, serum creatine kinase-MB (CK-MB) levels were significantly increased (*p* < 0.01) in the diabetic untreated group, suggesting cardiac injury.

Post-treatment with both low and high doses of Isorhamnetin, as well as metformin, significantly (*p* < 0.01) restored ALT and AST activities to near-normal levels, indicating improved cardiac function and reduced tissue damage. Similarly, CK-MB levels were significantly reduced (*p* < 0.01), indicating diminished myocardial injury and an overall cardioprotective effect of Isorhamnetin.

### Effects of Isorhamnetin on serum cardiac troponins and natriuretic peptide levels in STZ-Induced diabetic rats

**Fig. 6:**
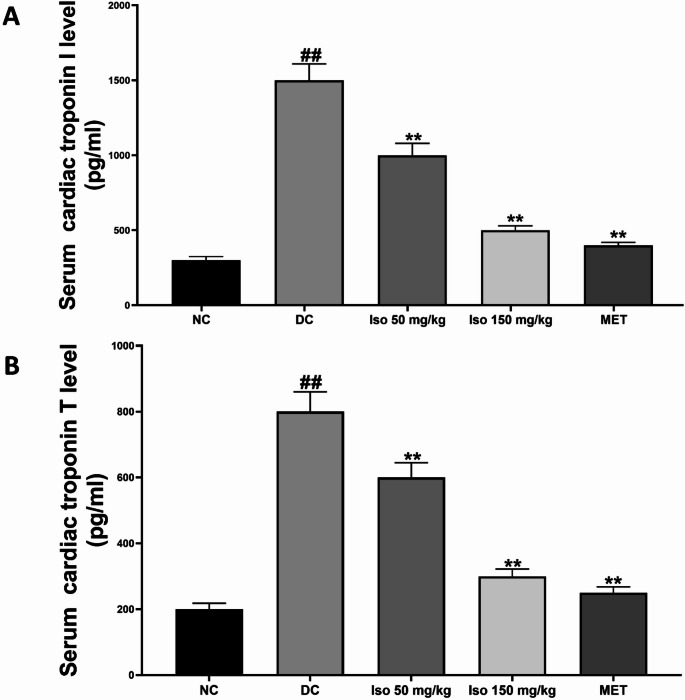
Isorhamnetin reduces cardiac troponin levels in streptozotocin-induced diabetic rats. Each value is a mean of eight determinations ± SD. ## *p <* 0.01 vs. NC, ** *p <* 0.01 vs. DC. NC: Normal Control, DC: Diabetic Control, MET: Diabetic rats administered 200 mg/kg of metformin

**Fig. 7:**
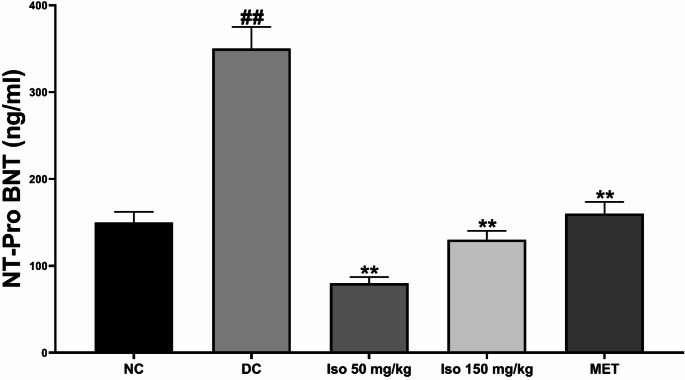
Isorhamnetin improved natriuretic peptide levels in streptozotocin-induced diabetic rats. Each value is a mean of eight determinations ± SD. ## *p <* 0.01 vs. NC, ** *p <* 0.01 vs. DC. NC: Normal Control, DC: Diabetic Control, MET: Diabetic rats administered 200 mg/kg of metformin

The serum level of cardiac biomarkers including cardiac troponins (I and T) and NT-ProBNP, a natriuretic peptide, in the experimental groups are shown in Figs. 6 and 7. Compared with normal control group, the content of cTnI and T as well as NT-ProBNP was significantly higher (*p* < 0.01) in diabetic model group (DC), which had more severe cardiac stress, damage and ventricular dysfunction.

The levels of these biomarkers were significantly lowered (*p* < 0.01) by treatment with Isorhamnetin at a dose of 40 or 80 mg/kg, both low and high doses indicating a possible inhibitory effect in different concentrations. This restoration was similar to those seen in the metformin-treated group, in which the levels of troponin and NT-ProBNP were reduced to near-normal levels compared with sham-operated rats. These results indicate that Isorhamnetin attenuates cardiac injury and ameliorates the heart function in diabetic conditions.

### Histological alterations in the heart of STZ-Induced diabetic rats and influence on Isorhamnetin

**Fig. 8:**
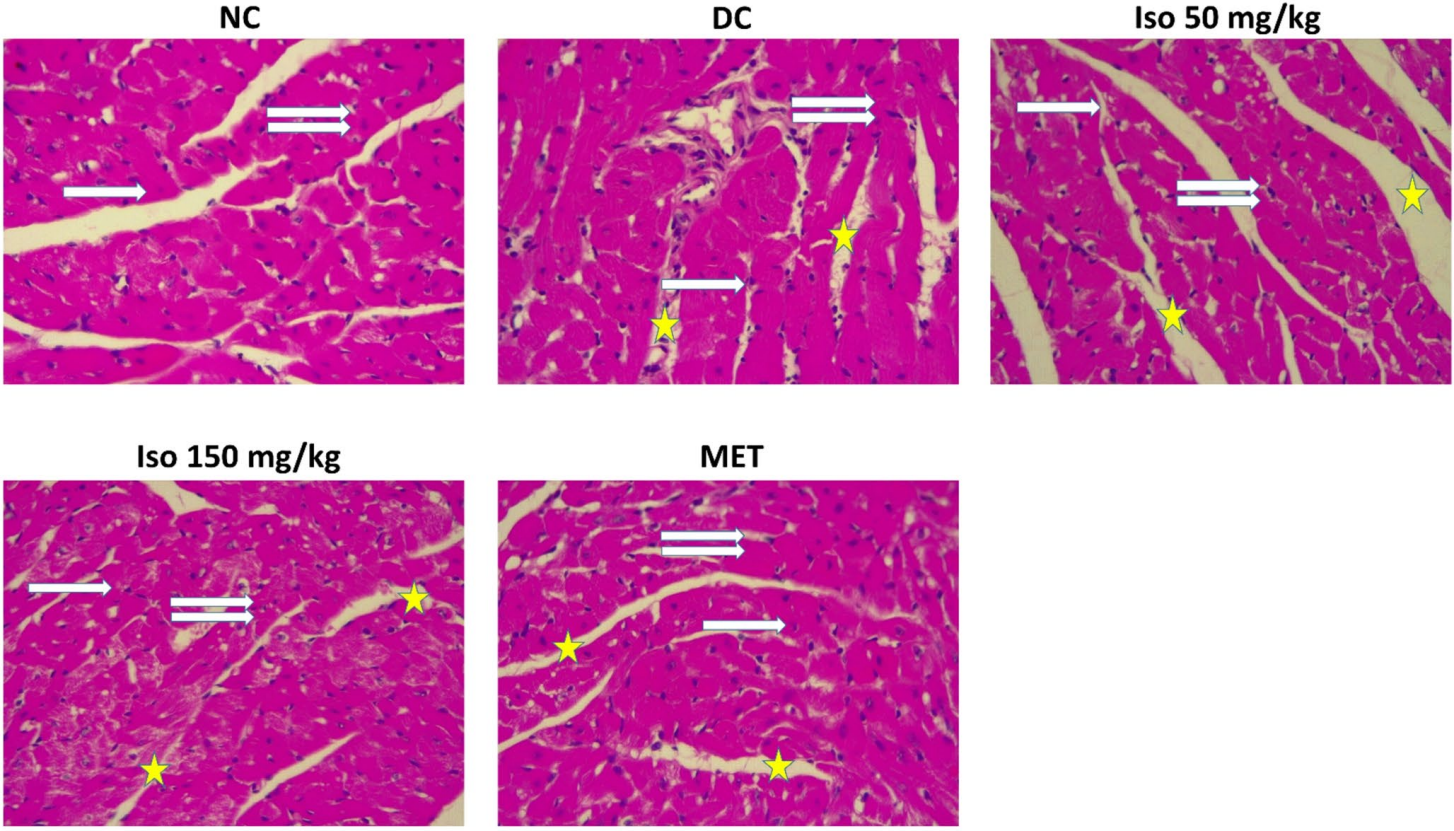
Isorhamnetin improved heart histoarchitecture examination in streptozotocin-induced diabetic rats. Tissues were stained with H&E. NC: Normal Control, DC: Diabetic Control, MET: Diabetic rats administered 200 mg/kg of metformin. NC: shows normal muscle fibers (one arrow) and normal centrally placed nuclei (two arrows); DC: shows vacuolated muscle fibers (one arrow), Degenerative nuclei (two arrows), and Congestion (star); Iso 50 mg/kg: shows normal muscle fibers (one arrow), normal and centrally placed nuclei (two arrows), few vacuolation (star); Iso 150 mg/kg: shows normal muscle fibers (one arrow), normal and centrally placed nuclei (two arrows); MET: shows normal muscle fibers (one arrow), normal and centrally placed nuclei (two arrows)

Photomicrographs in Fig. 8 depicted the histopathological changes in cardiac tissue of different experimental groups. Pathological changes such as moderate muscle fiber degeneration, vacuolization, nuclear degeneration and congestion of cardiac tissue reflected substantial structural injury related to diabetic cardiomyopathy in the untreated diabetic group (DC).

Remarkably, cardiac tissue architecture was drastically improved following treatment with both low and high doses of Isorhamnetin compared to metformin. The photomicrographs of treated diabetic rats revealed a decrease in number of vacuolated myofibres, better nuclear integrity and decreased congestion suggesting the near reversal of STZ induced damage. Especially the higher dose of Isorhamnetin, it was highly successful in causing a significant histological improvement similar to that inspection following metformin treatment, thereby advocating for its therapeutic potential to ameliorate diabetes-induced cardiac pathology.

## Discussion

This study aimed to determine the cardioprotective potential of Isorhamnetin in streptozotocin (STZ)-induced diabetic rats via studying its impact on oxidative stress markers, ATPase and phosphatases enzyme activities, cardiac gene expression as well as myocardial injury markers. The results indicate that Isorhamnetin, at low and high doses (i.e., 50 and 150 mg/kg), helps in substantially mitigating the diabetes-induced impairment of cardiac function as effectively such as the standard antidiabetic drug-metformin. Results suggest that Isorhamnetin may serve as a multi-targeted therapeutic agent for the treatment of diabetes-related cardiac complications.

The chronology of diabetes is particularly known for high level of oxidative stress that results in cellular injury and tissues malfunction [22]. Indeed, STZ-induced diabetic rats used in the current study presented elevated lipid peroxidation (verified by significant increase in malondialdehyde, MDA levels), confirming an increased oxidative damage. The result is in agreement with other studies demonstrated that the hyperglycemia-induced reactive oxygen species (ROS) generation could lead to a high level of MDA in diabetes related conditions [23].

Isorhamnetin treatment, at both low and high doses, resulted in substantial reduction of MDA levels. This decrease in MDA levels is consistent with the antioxidant properties of Isorhamnetin reported by other models of oxidative stress. For instance, Yang et al. (2014) and Wang et al. (2018) showed that Isorhamnetin is a powerful antioxidant which protected free radicals and lipid peroxidation [24, 25]. The antioxidative effects of Isorhamnetin are mainly associated with its hydroxyl groups in the chemical structure, which readily donate electrons to scavenge free radicals.

Apart from decreasing MDA levels, Isorhamnetin also recovered the activities of different important antioxidant enzymes such as Glutathione-S-transferase (GST), catalase (CAT), GPx and SOD in STZ-induced diabetic rats. This is mainly because it has been reported that diabetes impairs the functioning of these antioxidant enzymes largely by generating more reactive oxygen species (ROS) than can be counteracted by those cellular defenses against overstimulation with ROS [26]. This reduction in the CAT, SOD, GST and GPx activities is in agreement with previous reports demonstrating impaired antioxidant enzyme activity in the hearts of diabetic rats [27, 28]. Isorhamnetin scavenges the ROS and also up-regulates the endogenous antioxidant system. Indeed, in recent studies, Isorhamnetin could promote the expression of antioxidant enzymes which may be through the activation of Nrf2-signalling [24]. Nrf2 is the master transcription factor which governs the expression of antioxidant response elements, exerting a general role in inducing downstream synthesis of all antioxidant enzymes such as CAT, SOD and GPx [29]. Isorhamnetin can increase the activity of Nrf2, which might improve defenses against oxidative stress by activating innate antioxidant pathways in order to decrease oxidative stress and thus prevent oxidation of cardiac tissue [30].

Na^+^/K^+^-ATPase and Ca^2+^/Mg^2+^-ATPase are known to be cardiac ATPases, as well as Mg^2+^-ATPase, which have essential roles in establishing ion gradients that are necessary for the normal myocardial excitability, contractility  + and cellular homeostasis [31]. According to earlier reports, untreated diabetic rats showed a marked decrease in ATPase enzyme activities [32], showing that both the ion regulation and energy metabolism is deteriorated and these are the two important contributors of development of diabetic cardiomyopathy. In Diabetic individuals, the altered sodium channel function due to disturbed Na^+^/K^+^-ATPase can cause imbalance in sodium and potassium ion concentrations which causes increased cardiac excitability and associated susceptibility to arrhythmias [33].

Isorhamnetin treatment alleviated the down-regulation of these ATPase enzymes, which indicate its beneficial effects on cardiac ion transport and energy regulation. The antioxidant activity was suggested to be involved in the endothelial protective effects of isorhamnetin since oxidative damage has widely been reported to impair the functionality of membrane-bound enzymes like Na^+^/K^+^-ATPase [34]. Isorhamnetin could protect the structural integrity and function of these enzymes by reducing ROS levels during membrane peroxidation.

In addition, the anti-inflammatory effects of Isorhamnetin may also mediate its beneficial effects on ATPase activity. In diabetes, chronic inflammation also leads to malfunction of these ATPases by interfering by changing their expression and causing oxidative modifications [35]. Further, different studies have reported that Isorhamnetin suppresses the pro-inflammatory cytokines including TNF-α and interleukins [23]. The anti-inflammatory properties of Isorhamnetin might prevent the inflammatory damage of ATPase enzymes, improving their activity and heart health.

The study also examined the impact of Isorhamnetin on genetic biomarkers of p53 and VCAM-1 genes representing cellular stress and vascular inflammation, respectively. Since it has been documented in the literature that apoptosis and inflammation are increasing with diabetes [36], we took p53 and VCAM-1 as representative for these processes. This upregulation of p53 reflects a step in the progression which eventually would lead to apoptosis through mitrochonrdria signaling in the myocardium, and results in loss of cardiomyocytes and thus cardiac failure [37]. VCAM-1, by contrast, is important in leukocyte adhesion and the pathogenesis of vascular inflammation with a resultant contribution to diabetic atherosclerosis and endothelial dysfunction [38].

Treatment with isorhamnetin markedly reduced the expression of both p53 and VCAM-1, indicative of its anti-apoptotic and -inflammatory protective actions. The antioxidative effects of Isorhamnetin have a close connection with the downregulation of p53, and it is established that oxidative stress is powerful inducer for p53 expression in diabetic hearts [39]. Isorhamnetin probably lowers apoptosis by decreasing the activation of p53, a downstream signal for apoptotic cell death, which is in turn dependent on ROS reduction.

In vitro assays supported the anti-inflammatory effects of Isorhamnetin, which partially explained the downregulation of VCAM-1. Several previous studies have shown that isorhamnetin suppresses the nuclear NF-kappaB pathway, a key activator of expression of proi-nflammatory genes, and also up-regulation in levels of VCAM-1 [23]. Consequently, inhibition of NF-κB activation by Isorhamnetin suppresses the expression of adhesion molecules like VCAM-1 that promote vascular inflammation and it thus benefits endothelial function. Our results suggest that Isorhamnetin may have potential in alleviating vascular complications in diabetes through two intertwined mechanisms, i.e., by reducing the oxidative stress and inflammation.

The enzymes alkaline phosphatase (ALP) and acid phosphatase (ACP) catalyze those, the pro dephosphorylations processes and regulate cell metabolism and cell signalization. The diabetic heart under stress similarly exhibited abrogated PP2A activation with reduced cardioprotective phosphatase activities, reflecting an apparent dysfunction of the enzyme that could conceivably contribute to altered metabolic and tissue damage observed in these hearts [40]. These include the vascular protective effects of ALP, with impaired activity linked to endothelial dysfunction and accelerated vascular calcification in diabetes [41].

Treatment with isorhamnetin greatly increased the levels of ALP and ACP, suggesting a protecting role on metabolic and signaling activities in cell. This increase in expression of genes for the phosphatase activity may be associated with the decrease in oxidative stress and inflammation overall observed by Isorhamnetin, as both oxidative damage and inflammatory signaling are capable of impacting activity of phosphatases negatively [42]. Phosphatase activity may be preserved by Isorhamnetin, and in this way it might contribute to the preservation of normal cellular signaling and prevent vascular calcification and tissue damage in diabetic hearts.

Because CK-MB, ALT, AST and cardiac troponins (cTnI and cTnT) are specific markers of cardiomyocyte injury in diabetic rats, there is myocardial damage caused by hyperglycemia-induced effects [43]. Despite the fact that high CK-MB and troponins reflect myocardial cell injury, these indexes have also been exploited when seeking for cardiac damage biomarkers in diabetic patients [44].

Levels of these cardiac injury markers after isorhamnetin treatment were also apparently decreased, implying its cardioprotective effects. The antioxidants, anti-apoptotic and anti-inflammatory effects of Isorhamnetin may explain this decrease in CK-MB and troponins. Isorhamnetin, by lowering levels of oxidative stress and inflammation, as a result, it has turned out to be protective against myocardial cell injury and alleviate the release of these injury markers.

Treatment with Isorhamnetin also significantly decreased NT-proBNP levels, a marker for cardiac wall stress and heart failure. In diabetic cardiomyopathy, increased levels of NT-proBNP have been widely detected which indicates higher cardiac workload and ventricular dysfunction [45]. The decrease of NT-proBNP after Isorhamnetin treatment suggests an attenuation of the cardiac function impairment and reduced ventricular stress. Since Isorhamnetin can improve ATPase activity, result in ion homeostasis and myocardial contractility — these changes reflected the removal ventricular arrhythmia of Isorhamnetin.

In addition, histopathological examination of cardiac tissues provides evidence that Isorhamnetin exert beneficial effects. Apathological histology, including muscle degeneration, vacuolation, nuclear degradation, congestion was observed in diabetic control rats. In the pathological analyses, these changes are also reflected in elevated indices of oxidative damage, inflammation, and compromised cellular integrity, hallmarks of diabetic cardiomyopathy [46].

The improvement observed in the histological picture of the cardiac tissue was remarkable after treatment with Isorhamnetin, especially at high doses as indicated by less vacuolization and better nuclear integrity beside a decreased congestion. These results are in agreement with precedent studies demonstrating that Isorhamnetin can decrease the insult to tissues and improve the structural integrity of them under oxidative and inflammatory stress [47].

Isorhamnetin demonstrated its cardioprotective effect via decrease in oxidative stress, increase in level of antioxidant defenses up regulation of ATPase and phosphatase activities down-regulation of the pro-apoptotic and pro-inflammatory genes reduction of myocardial injury markers. The results indicate that Isorhamnetin is an interesting multi-target drug for the treatment of diabetic cardiac complication.

This effect of Isorhamnetin on antioxidant enzyme activities and ROS levels suggests potential benefits against oxidative stress, which are strong inducers of the diabetic complications. Moreover, enhancement of ATPase and phosphatase activities indicated the recovery of energy metabolism and signaling in cardiac that may contribute to improving improve heart function. It also suppressed p53 and VCAM-1 expression indicating that it is involved in apoptosis reduction as well as vascular inflammation, crucial factors underlying diabetic cardiovascular complications progression.

These data are in keeping with prior work which corroborated the cardioprotective effects of flavonoids and other naturally occurring compounds in diabetic states. Similarly, other flavonoids such as quercetin, kaempferol and rutin have displayed the ability to reduce oxidative stress, inflammation and myocardial injury in diabetes [48, 49], hence prove possible Isorhamnetin is one of a number of natural agents with potential to act in a cardioprotective capacity on its own or as part of its class.

## Conclusion

In summary, the novelty of this present study is that we emphasized the cardioprotective effects of Isorhamnetin in STZ-induced diabetic rats. Isorhamnetin mitigates oxidative stress, enhances ATPase and phosphatase activities, alterations in pro-apoptotic/pro-inflammatory gene expressions and improves myocardial injury markers. This is a result of an ontology of antioxidative, antiapoptotic and anti-inflammatory mechanisms that make it an interesting candidate to address cardiac complications induced by diabetes. More mechanistic investigations, to identify the cellular targets/pathways of Isorhamnetin and human studies, are warranted for its potential therapeutic use in treatment of diabetes-related CVD.

## Data Availability

Data associated with this publication will be available from the corresponding author upon request.
